# Improves in external and internal egg quality of Japanese quail (*Coturnix coturnix japonica*) by giving lactic acid bacteria as alternative antibiotic growth promoter

**Published:** 2019-10

**Authors:** Widya Paramita Lokapirnasari, Anam Al Arif, Soeharsono Soeharsono, Anisah Fathinah, Rifqi Najwan, Hana Cipka Pramuda Wardhani, Nabil Fariz Noorrahman, Khoirul Huda, Nuria Ulfah, Andreas Berny Yulianto

**Affiliations:** 1 Department of Animal Husbandry, Faculty of Veterinary Medicine, Universitas Airlangga, Surabaya, Indonesia; 2 Department of Anatomy Veteriner, Faculty of Veterinary Medicine, Universitas Airlangga, Surabaya, Indonesia; 3 Department of Animal Husbandry, Magister of Veterinary Agribusiness, Faculty of Veterinary Medicine, Universitas Airlangga, Surabaya, Indonesia

**Keywords:** *Lactobacillus casei*, *Lacrobacillus rhamnosus*, Japanese quail, External and internal egg quality

## Abstract

**Background and Objectives:**

An experiment was designed to determine the effect of using lactic acid bacteria as alternative antibiotic growth promoters on external and internal quality of egg’s *Coturnix coturnix japonica*.

**Materials and Methods:**

*Coturnix coturnix japonica* (n=240, 14 weeks of age) were randomly distributed into six treatment groups. The treatments were P0 (free antibiotic feed), P1 (free antibiotic feed with 1 gram antibiotic growth promoters (AGP)/100kg feed), P2 (free antibiotic feed with 5 gram probiotic/100kg feed), P3 (free antibiotic feed with 10 grams probiotic/100kg feed), P4 (free antibiotic feed with 5 gram probiotic/200L drinking water), and P5 (free antibiotic feed with 10 gram probiotic/200L drinking water). Probiotic contained *Lactobacillus casei* (*L. casei*) and *Lactobacillus rhamnosus* (*L. rhamnosus*) culture (1.2 × 10^8^ CFU/gram). To assess the quality parameters, twenty eggs were randomly collected from each treatment at the end of the experimental period, and the data were analysed using one way Anova.

**Results:**

Results of the external quality indicated that egg’s weight, length, and width, along with the shell weight and thickness were significantly different (P<0.05) after treatment. Likewise, the results of internal egg quality indicated that yolk color, height, width, and length, together with the albumen height, width, length, index and haugh unit were significantly different (P <0.05) after treatment.

**Conclusion:**

It was concluded from this research that dietary supplementation with probiotic which contains *L. casei* and *L. rhamnosus* could be used in laying Japanese quail with benefit on external and internal egg quality.

## INTRODUCTION

The population of Japanese quail, *Coturnix coturnix japonica* in Indonesia has increased, based on data from the Director of Animal Husbandry and Animal Health (2016) populations of quail in Indonesia. A figure of 12, 692, 213 was recorded in 2014, 13, 781, 918 in 2015 and 13, 932, 649 in 2016. The demands for quail meat and eggs therefore increase as much as consumer attraction (1).

Antibiotic growth promoters (AGP) have been widely applied to improve animal products. This practice has raised concerns for the emergence of antibiotic resistant that could potentially spread to humans (2). Probiotics have the potential of becoming an AGP alternative. *Lactobacillus* strains can enhance growth performance (3), improve meat quality (4), increase immune response (5), and prevent some avian diseases (6, 7). Egg is one source of protein which consumers believe to have low prices among animal products. The prices of eggs, including nutritional eggs are substantially influenced by the internal and external characteristics of the egg (8, 9).

Giving probiotic in poultries improves the utilization of nutrients and feed conversion ratio, enhances feed efficiency, and preserves the health status of the animals (10). Mechanisms of probiotics by enhancing the development of non-pathogenic facultative anaerobic bacteria and Gram positive bacteria forming hydrogen peroxide and lactic acid as well as suppressing the growth of intestinal pathogens (11, 12), strengthening the intestinal immune response, improving feed conversion efficiency and including alteration in intestinal flora. The intestinal bacterial populations have an important physiological and pathological effect on the host (13, 14). A stable intestinal microflora can protect the host from pathogen colonization by producing antimicrobial bacteriocins, by competing for epithelial binding sites and nutrients (15) and improving utilization and nutrient availability (16–18).

This study discovers the possible synergistic effect of probiotics containing *Lactobacillus casei* and *Lactobacillus rhamnosusas* alternative antibiotic growth promoters that can be beneficial for the improvement of both the external and internal quality of egg’s *Coturnix coturnix japonica*.

## MATERIALS AND METHODS

A total of 240 laying Japanese quail hens, which had been in production for 14 weeks, were randomly distributed into six treatments, having four replicates of 10 quails in each in a completely randomized design, with the following treatments: P0: free antibiotic feed (without probiotic and AGP, as control); P1: free antibiotic feed + AGP 1 gram/100kg feed; P2: free antibiotic feed + Probiotic 5 gram/100 kg feed; P3: free antibiotic feed + Probiotic 10 gram/100vkg feed; P4: free antibiotic feed + Probiotic 5g/200vL water; P5: free antibiotic feed + Probiotic10g/200L water. Ration formulation for *Coturnix coturnix japonica* consists of crude protein min 17%, crude fiber max 7%, extract ether max 7%, ash max 14%, Ca 2.5–3.5%, Metabolizable energy min 2700kkal/kg (SNI 01-3907-2006). The isolates were *Lactobacillus casei* and *Lactobacillus rhamnosus* collected by Widya Paramita Lokapirnasari, Department of Animal Husbandry, Faculty of Veterinary Medicine, Universitas Airlangga.

One gram of AGP (P1), five grams of probiotic (P2) and ten grams of probiotic (P3) were mixed with 100 kg of feed in concentration 1.2 × 10^5^ CFU/gram, dissolved in 995 mL and 990 mL of water (free chlorine and other antiseptics), and then allowed to stand for 24 hours without aeration. A total of one liter probiotic solution was sprayed evenly into 100 kg of feed which was then left to dry so the probiotics absorbed well in it, making it ready to be fed. All Japanese quails were reared in wire batteries under the same environmental conditions. Water and feed were available, while the ad libitum and light regimen was about 16 h of light.

External and internal quality of the egg were estimated using following formula (19): W=Egg weight (g); L= egg length (cm); Wi=egg width (cm); SW= shell weight (g); ST= shell thickness (cm); AL=albumen length (mm); AW= albumen width (mm); AH= albumen height (mm); AI=albumen index (%)=Albumenheight (mm)/[(Albumenlength (mm)+ albumen width (mm)]/2)x100; Haughunit (HU)=100log (H+7.57-1.7W^0.37^)

### Data collection

Egg quality measurements were taken using an average of 20 eggs from each treatment. The internal quality test was performed by carefully separating the eggs on a flat glass surface. The lengths and widths of the egg white and yolk were measured using vernier caliper. The heights were measured using a spherometer.

### Statistical analysis

All data were analysed using the Analysis of Variant (ANOVA) procedure in a completely randomized design with the SPSS software for Windows. Furthermore, the differences among all treatments were separated by Duncan’s multiple range tests. Results expressed as p<0.05 were considered statistically significant.

## RESULTS

The results of egg weight, egg length, egg width, shell weight and shell thickness showed that there were significant differences between treatments (p<0.05) compared to control. The egg weight of P0 treatment was significantly different (p<0.05) with P1, P3, P4 and P5, but P0 treatment was not significantly different (p>0.05) with P2 treatment. P1 treatment showed significant differences (p<0.05) with P3, P4 and P5 treatment. P2 treatment was not significantly different (p>0.05) with P0 and P1 treatment, but P2 was significantly different (p<0.05) with P3, P4 and P5 treatments. The highest egg weight results were found in treatment P5, P4 and P3, while the lowest egg weight was found in P0 treatments.

The result egg length of P0 treatment was significantly different (p<0.05) with P1, P3, P4 and P5, but P0 treatment was not significantly different (p>0.05) with P2 treatment. P1 treatment showed significant differences (p<0.05) with P2 and P4 treatment, but not significantly different (p>0.05) with P3 and P5 treatment. P2 treatment was not significantly different (p>0.05) with P0 treatment, but P2 was significantly different (p<0.05) with P1, P3, P4 and P5 treatments. The highest egg length results were found in treatment P5, P4 and P3, while the lowest egg length was found in P0 and P2 treatments.

The results of egg width was significant differences between treatments (p<0.05) compared to control. The egg width of P0 treatment was significantly different (p<0.05) with P2, P3 and P5, but P0 treatment was not significantly different (p>0.05) with P1 and P4 treatment. P1 treatment showed significant differences (p<0.05) with P2, but not significantly different (p>0.05) with P0,P3, P4 and P5. The treatment of P2 was significantly different (p<0.05) with P0, P1, P3, P4 and P5 treatments. The highest egg width results was found in treatment P2, while the lowest egg width was found in P0, P1 and P4 treatments.

The results of shell weight was significant differences between treatments (p<0.05). The shell weight of P0 treatment was significantly different (p<0.05) with P1, P3 and P5, but P0 treatment was not significantly different (p>0.05) with P2 and P4 treatment. P1 treatment showed significant differences (p<0.05) with P0, P2 and P4, but P1 was not significantly different (p>0.05) with P3, and P5. The treatment of P2 was significantly different (p<0.05) with P1 and P5 treatments. The highest shell weight results was found in treatment P5, while the lowest shell weight was found in P0, P1 and P4 treatments.

The results of shell thickness was significant differences between treatments (p<0.05). The shell weight of P0 treatment was significantly different (p<0.05) with P1, P2, P4 and P5, but P0 treatment was not significantly different (p>0.05) with P3 treatment. P1 treatment showed significant differences (p<0.05) with P0 and P3, but P1 was not significantly different (p>0.05) with P2 and P5. The treatment of P2 was significantly different (p<0.05) with P0 and P3 treatments. The highest shell thickness results was found in treatment P0 and P3, while the lowest shell thickness was found in P4, P2 and P5 treatments. The effects of probiotic supplementation on external egg quality in Japanese quail are shown in Table 1.

**Table 1:** Effect of supplementation *L. casei* and *L. rhamnosus* on external egg quality of Japanese quail

**Treatment**	**Egg weight (g/egg)**	**Egg length (cm)**	**Egg width (cm)**	**Shell weight (g)**	**Shell thickness (cm)**
P0	10.85^a^ ± 0.45	3.19^a^ ± 0.16	2.47^a^ ± 0.07	1.37^a^ ± 0.19	0.055^c^ ± 0.01
P1	11.32^b^ ± 0.23	3.26^b^ ± 0.03	2.51^ab^ ± 0.02	1.54^c^ ± 0.07	0.042^b^ ± 0.00
P2	10.98^ab^ ± 0.63	3.21^a^ ± 0.09	3.02^c^ ± 0.27	1.41^ab^ ± 0.12	0.040^ab^ ± 0.00
P3	11.68^c^ ± 0.39	3.31^bc^ ± 0.05	2.57^b^ ± 0.02	1.47^bc^ ± 0.12	0.053^c^ ± 0.01
P4	11.68^c^ ± 0.48	3.36^c^ ± 0.05	2.54^ab^ ± 0.06	1.37^a^ ± 0.07	0.038^a^ ± 0.00
P5	11.71^c^ ± 0.88	3.31^bc^ ± 0.13	2.58^b^ ± 0.05	1.54^c^ ± 0.09	0.040^ab^ ± 0.01

(a, b, c) Means in the same column with the different superscript are significantly different at (p<0.05).

The effects of probiotic supplementation on internal egg quality (egg yolk) in Japanese quail are shown in Table 2.

**Table 2:** Effect of supplementation *L. casei* and *L. rhamnosuson* internal egg quality of Japanese quail

**Variable**	**Treatment**					

	**P0**	**P1**	**P2**	**P3**	**P4**	**P5**
Albumen length (mm)	8.56^a^ ± 0.51	9.44^b^ ± 0.88	10.31^c^ ± 0.93	11.32^d^ ± 0.39	11.54^d^ ± 0.75	10.69^c^ ± 0.85
Albumen width (mm)	6.56^a^ ± 0.25	7.20^b^ ± 0.67	7.70^bc^ ± 1.40	8.12^c^ ± 0.63	8.01^c^ ± 0.70	8.64^d^ ± 0.71
Albumen height (mm)	1.74^d^ ± 0.51	1.47^a^ ± 0.88	1.56^ab^ ± 0.93	1.46^a^ ± 0.39	1.62^b^ ± 0.75	1.52^ab^ ± 0.85
Albumen index (%)	2.3^c^ ± 0.05	1.7^b^ ± 0.02	1.7^b^ ± 0.02	1.5^a^ ± 0.01	1.6^ab^ ± 0.01	1.6^ab^ ± 0.01
HU	70.97^d^ ± 3.34	68.12^ab^ ± 0.78	69.37^c^ ± 1.29	67.60^a^ ± 0.82	68.97^bc^ ± 0.99	68.07^ab^ ± 1.23

a, b, c) Means in the same column with the different superscript are significantly different at (p<0.05).

The results of albumen length, width, height, albumen index and haugh unit (HU) demonstrated that there were significant differences between treatments (p<0.05). The albumen length of P0 treatment was significantly different (p<0.05) with P1, P2, P3, P4 and P5. P1 treatment showed significant differences (p<0.05) with P0, P2, P3, P4 and P5 treatment. P2 treatment was significantly different (p<0.05) with P0, P1, P3, P4 and P5 treatments. The treatment of P3 was not significantly different (p>0.05) with P4. The highest albumen length results were found in treatment P4 and P3, while the lowest albumen length was found in P0 treatments.

The albumen width of P0 treatment was significantly different (p<0.05) with P1, P2, P3, P4 and P5. P1 treatment showed significant differences (p<0.05) with P0, P3, P4 and P5 treatment, but it was not significantly different (p>0.05) with P2. P2 treatment was significantly different (p<0.05) with P0 and P5 treatments, but it was not significantly different (p>0.05) with P1, P3 and P4. The treatment of P3 was not significantly different (p>0.05) with P4 and P2. The highest albumen width results were found in treatment P4, while the lowest albumen width was found in P0 treatments.

The albumen height of P0 treatment was significantly different (p<0.05) with P1, P2, P3, P4 and P5. P1 treatment showed significant differences (p<0.05) with P0 and P4 treatment, but it was not significantly different (p>0.05) with P2, P3 and P5. P2 treatment was significantly different (p<0.05) with P0 treatment but it was not significantly different (p>0.05) with P1, P3, P4 and P5. The treatment of P3 was not significantly different (p>0.05) with P1, P2, and P5, but it was significant differences (p<0.05) with P0 and P4. The highest albumen height results were found in treatment P0, while the lowest albumen height was found in P1 and P3 treatments.

## DISCUSSION

### External eggs quality

The effects of *Lactobacillus casei* and *Lactobacillus rhamnosus* supplementation on external egg quality in Japanese quail are shown in Table 1. The results obtained in this research showed that probiotic supplementation to the quail could improve the external quality of quail eggs. The results showed that the egg weight, length, and width, along with the shell weight and thickness were significantly different (p<0.05) when compared with the controls. The supplementation of 10 gram probiotic/100 kg feed (P3), 5 and 10 grams probiotic/200L water (P4, P5) exhibited the same effect higher than the other treatments to egg weight. The mean value was 11.68 to 11.71 g/egg. Egg length (p<0.05) increased significantly more than the control. The mean values were 3.31 to 3.36 cm for egg length in quails supplemented with 10 gram probiotic/100 kg feed, 10 and 5 gram probiotic/200L water, respectively. Egg width (p<0.05) increased significantly higher than the other treatments. The highest egg width was 3.02 cm in quails supplemented with 5 gram probiotic/100 kg feed. Shell weight (p<0.05) increased significantly higher than the control. The mean value was 1.47 to 1.54 g for shell weight in quails supplemented with 10 grams probiotic/200L water and 10 grams probiotic/100 kg feed respectively. The group receiving no probiotic and 10g/100 kg feed attained the highest shell thickness. Shell thickness in that group was found to be 0.055 cm. The supplementation of probiotics in Japanese quails did not improve the shell thickness (20) and no significant differences were observed in feed consumption and most of the studied egg quality (egg, yolk and albumen weights, shell percentage, egg specific gravity, albumen index, and yolk index) among laying quails fed with *Aspergillus awamori* supplemented as probiotic (21).

In this study, supplementation *Lactobacillus casei* and *Lactobacillus rhamnosus* had a significant effect on egg weight. Some factors that cause variation of egg weight include the natural pattern of its production, feed, management, strain, age, feed consumption, disease (22), and other factors related to genetics. Several studies which made use of probiotic cultured indicated that probiotics had an effect on laying hens (23–24). The obtained results in this research, the egg weight of Japanese quail by giving probiotics was 10.98–11.71 g. It appears to indicate that Japanese quail egg of 10.51–12.50 g are the best for hatching (25). The survival rate of quail was significantly affected by the main effects of hatching egg weight (26).

### Internal eggs quality

The physical characteristics of eggs which includes the internal quality, and this comprises of the length, width, and height, the albumen length, width, height, and index, together with the haugh unit showed a value in the treatment group (p<0,05) different from the control group. The Haugh Unit value was lower in group P5 with 10 grams of probiotic through water when compared to controls and other treatment groups, as presented in Table 2.

The results of the internal egg yolk quality revealed that the yolk length, width, height and index were significantly different (p<0.05) when compared with the controls (Table 2). The supplementation of 5 gram probiotic/200L water (P4) and AGP 1 gram/100 kg feed (P1) showed the yolk length and width (p<0.05) significantly increase higher than in the control treatment. The high values were 11.32 mm (P3) and 11.54 mm (P4) for the length and 8.64 mm (P5) for the width in quails supplemented with 10 gram probiotic/100 kg feed. The lowest value was 8.56 mm in length and 6.56 mm for width in quails without supplementing with probiotic (P0). Yolk height (p<0.05) significantly increased higher than those of the other treatments. The highest height was 1.62 mm in quails supplemented with 5 gram probiotic/200L water (P4) and no significant difference with 10 gram probiotic/200 L water (P5).

Differences in all egg quality affected by species, genotype, age, feeding, and management (27). The yolk index and haugh units are important internal characteristics in the poultry industry (28). Haugh unit (HU) is a way of defining the degree of freshness of eggs using the thickness of the albumen. The determining pattern by using the break out method, the height or thickness of the measured albumen compared with the egg weight. A thicker egg albumen indicates a fresher egg. The supplementation of probiotic both in feed and water give a significant impact to feed conversion ratio, feed consumption, enhance feed efficiency, and quail day production (29). Lactic acid bacteria (LAB) are generally recognized as safe microorganisms and the majority of probiotics (30).

In conclusion, *L. casei* and *L. rhamnosus* as probiotic supplementations offer a feasible way to improve external and internal egg quality of Japanese quail (*Coturnix coturnix japonica*).
